# Association Between Beta-Carotene Supplementation and Mortality: A Systematic Review and Meta-Analysis of Randomized Controlled Trials

**DOI:** 10.3389/fmed.2022.872310

**Published:** 2022-07-19

**Authors:** Graziamaria Corbi, Sawan Ali, Mariano Intrieri, Sergio Modaferri, Vittorio Calabrese, Sergio Davinelli, Giovanni Scapagnini

**Affiliations:** ^1^Department of Translational Medical Sciences, University of Naples Federico II, Naples, Italy; ^2^Department of Medicine and Health Sciences, University of Molise, Campobasso, Italy; ^3^Department of Biomedical and Biotechnological Sciences, University of Catania, Catania, Italy

**Keywords:** mortality, meta-analysis, randomized controlled trials, aging, beta-carotene

## Abstract

**Background:**

Aging is a phenomenon universally involving all organisms, genetically determined, and epigenetically influenced by the environment. Numerous observational studies have shown the positive impact of non-pharmacological approaches started in younger age on chronic conditions affecting the elderly health and survival. This meta-analysis aimed to investigate the effect of beta-carotene on the total and cause-specific mortality as reported by randomized controlled trials (RCTs).

**Methods:**

We searched Medline, Scopus, Web of Science, and CENTRAL Cochrane from inception to September 2021. Studies were eligible if enrolled adults with any health condition, compared beta-carotene supplements at any dose with placebo or no intervention, provided information on deaths from any cause, and were RCTs, in English. The risk of bias was assessed by the Cochrane risk of bias tool and the GRADE. Risk ratios and their 95% confidence intervals were used and a *P*-value less than 0.05 was considered statistically significant.

**Results:**

Among 3,942 articles searched, 44 articles on 31 RCTs, which included 216,734 total subjects, 108,622 in beta-carotene supplement groups, and 108,112 in the placebo or no-intervention groups, were involved in the final analyses. In a random-effects meta-analysis of all 31 trials, beta-carotene supplements were found to have no preventive effect on mortality (risk ratio 1.02, 95% confidence interval 0.98–1.05, *I*^2^ = 42%). Further, the analysis showed no preventive effect on cancer, cardiovascular, cerebrovascular, and other mortality causes. Instead, beta-carotene supplementation significantly increased the risk of lung cancer mortality (RR 1.14, 95% CI 1.02, 1.27, *I*^2^ = 3%) but decreased the risk of human immunodeficiency virus-related mortality (RR 0.55, 95% CI 0.33, 0.92, *I*^2^ = 0).

**Conclusion:**

More studies should be performed to better define the role of beta-carotene on survival, to confirm or deny our results. Therefore, the possible beneficial or harmful effects of the beta-carotene supplementation on mortality must not be overstated.

**Systematic Review Registration:**

[https://www.crd.york.ac.uk/prospero/display_record.php?RecordID=259354], identifier [CRD42021259354].

## Introduction

Aging is a phenomenon universally involving all organisms, genetically determined, epigenetically influenced by the environment, and characterized by a progressive decline of physiological function, mainly the cardiovascular and metabolic profile, leading to death. Numerous observational studies have shown the positive impact of non-pharmacological approaches started in younger age on chronic conditions affecting the elderly health and survival (1–4).

Nutrition is a modifiable lifestyle factor that has been consistently associated with various aspects, including greater adherence to healthy dietary patterns, the intake of specific nutrients, or the consumption of specific foods (5).

Beta-carotene is a fat-soluble phytochemical found naturally in yellow/orange and green leafy plants, and also produced by some microorganisms (6). It is a single homolog of nearly 600 known carotenoids, several of which can be converted into vitamin A and occur as cis-trans forms at a varying ratio (7, 8). As the main carotenoids, beta-carotene can be metabolized into bioactive retinol and other beta-carotene compounds essential for maintaining homeostasis and human physiology (9). Several studies reveal that the beta-carotene is a potent antioxidant, able to function against oxidative stress, maintaining health, and preventing diseases such as cancer and cardiovascular disease (CVD) (10–15). Observational evidence also suggests that a high dietary intake of beta-carotene is associated with a reduced risk of cancer and CVD (16). Moreover, serum beta-carotene has also been inversely correlated with systemic inflammation and insulin resistance (17, 18). However, there is also evidence that beta-carotene may possess a pro-oxidant property and act as a cocarcinogen (19).

Several studies, including meta-analyses, assessing the health effects of beta-carotene showed inconsistent results in humans. Although there have been mixed results for the risk of mortality from cancer (20–22), several observational studies indicated that individuals with a high dietary intake or high circulatory levels of beta-carotene have a lower risk of all-cause (21) and CVD mortality (20, 23, 24). According to a meta-analysis of prospective studies, dietary or circulating beta-carotene has an inverse association with total mortality (25). In addition, in another recent dose-response meta-analysis of observational studies, higher circulating concentrations of beta-carotene were significantly associated with a lower risk of CVD mortality, whereas higher dietary intake of beta-carotene did not appear to have protective effects (26). As a supplement, the findings were inconsistent. Large controlled trials reported either no benefits or unpredicted adverse effects of beta-carotene supplementation, including increased lung cancer incidence and mortality among subjects exposed to asbestos and tobacco (27–30). In these treatment trials, beta-carotene also led to a small but significant increase in CVD and augmented total mortality. In 2012, a meta-analysis of RCTs was conducted by the Cochrane group. In trials with a low risk of bias, the results demonstrated that beta-carotene used singly or in combination with other antioxidants significantly increases overall mortality (31). Furthermore, the same review group performed a meta-regression analysis and reported significant effects of the dose of beta-carotene on mortality (32).

There has been substantial attention to the health effects of beta-carotene, and a systematic review and meta-analysis of the association between beta-carotene supplementation and all-cause mortality in RCTs have already been reported (31). However, the last analyses referred to data available until 2012, and a better and more updated understanding of the beta-carotene-mortality association to examine cause-specific mortality is needed. Therefore, this meta-analysis investigates the association between beta-carotene supplementation and the risk of cause-specific mortality among population subgroups in RCTs, including the most recent results in the literature.

## Materials and Methods

This study was performed following Preferred Reporting Items for Systematic Reviews and Meta-Analysis (PRISMA) 2020 (33). The protocol for this review was registered on PROSPERO (CRD42021259354).

### Inclusion and Exclusion Criteria

Studies were eligible if they enrolled adults (age ≥ 18) with any health condition; if they compared beta-carotene supplements at any dose with placebo or no intervention, provided information on deaths from any cause; and if they were randomized controlled trials (RCTs). On the contrary, we excluded studies if all the participants received beta-carotene; if they included pregnant women or critically ill patients; and if they used beta-carotene analogs.

### Search Strategy

We searched four databases: Medline, Scopus, Web of Science, and the Cochrane Central Register of Controlled Trials (CENTRAL) of the Cochrane library, from inception to September 2021. We also checked the bibliography of identified studies and systematic reviews to increase the search for relevant articles. We applied English language restriction. No restriction on the type of publication was used. We selected the following keywords for the literature search: “carotenoid*,” or “beta-carotene,” or “b-carotene” and “mortality,” or “death.” At the same time, similar queries were, respectively, used for controlled vocabulary search: “beta-carotene” [Mesh] AND “mortality” [Mesh], INDEX TERMS “beta-carotene” AND “mortality.”

### Study Selection and Data Extraction

After removing duplicates with reference management software EndNote X9 (Clarivate Analytics, Philadelphia, PA, United States), Two raters screened the title/abstract of articles independently. Potentially eligible articles were then accessed in full. Divergences between raters on article eligibility were resolved by a third rater, who screened the studies independently (100% consensus on article eligibility was reached). A data extraction spreadsheet was then developed, and the information from the included studies was extracted and tabulated. When RCTs had more than two arms, data from the separate treatment arms were pooled. The following data were extracted: study name (along with the year of publication), country, study characteristics (participant number, age, gender, health status, and study design), treatment duration/follow-up period, intervention and dosage, mortality causes, and the amount of death/number of participants in each intervention group.

### Study Quality Assessment

The quality of all included trials was assessed using the Cochrane Collaboration risk of bias tool (34). The Cochrane risk of bias tool is made up of 7 components: (1) sequence generation, (2) allocation sequence concealment, (3) blinding of participants and personnel, (4) blinding of outcome assessment, (5) incomplete outcome data, (6) selective outcome reporting, and (7) other bias. Moreover, we also performed the GRADEpro GDT (GRADEpro Guideline Development Tool Software (35) assessment for the quality of evidence.

### Statistical Analyses

We performed statistical analyses using RevMan (version 5.3.3; The Cochrane Collaboration) and the meta package in R Software, version 4.0.3 (R Foundation for Statistical Computing, Vienna, Austria), and the interface R-Studio version 1.4.1717 (R studio, PBC, Boston, MA, United States). We used risk ratios and their associated 95% confidence intervals to assess outcomes and considered a *P*-value less than 0.05 to be statistically significant. We assessed heterogeneity using the *I*^2^-test (34). We used random-effects models for our analysis and the possibility of small study effects was assessed qualitatively by a visual estimate of the funnel plot and quantitatively by calculation of the Egger and Begg’s tests (36).

We evaluated the effects of beta-carotene supplements according to mortality cause (cancer mortality, CVD mortality, cerebrovascular disease mortality, and mortality from other causes). Besides, we performed several additional subgroup analyses to test interactions according to: the number of participants (≥1,000 and <1,000, by using the median value for stratification), the number of events (≥100 and <100 by using the median value for stratification), the gender (men, women, and both), the mean age (≥65 and < 65 years to evaluate the aging effect), the beta-carotene dose (>20 and <20 mg/day by using the median value for stratification), the length of follow-up (at least four years and less than four years, by using the median value for stratification), the intervention (beta-carotene singly and beta-carotene combined with vitamins, minerals, or other interventions), the participant health status (healthy and unhealthy), and the control group (placebo and no intervention) in all the included trials. Moreover, a subgroup analysis was also performed by country (Supplementary Figure 7).

## Results

### Study Selection

**FIGURE 1:**
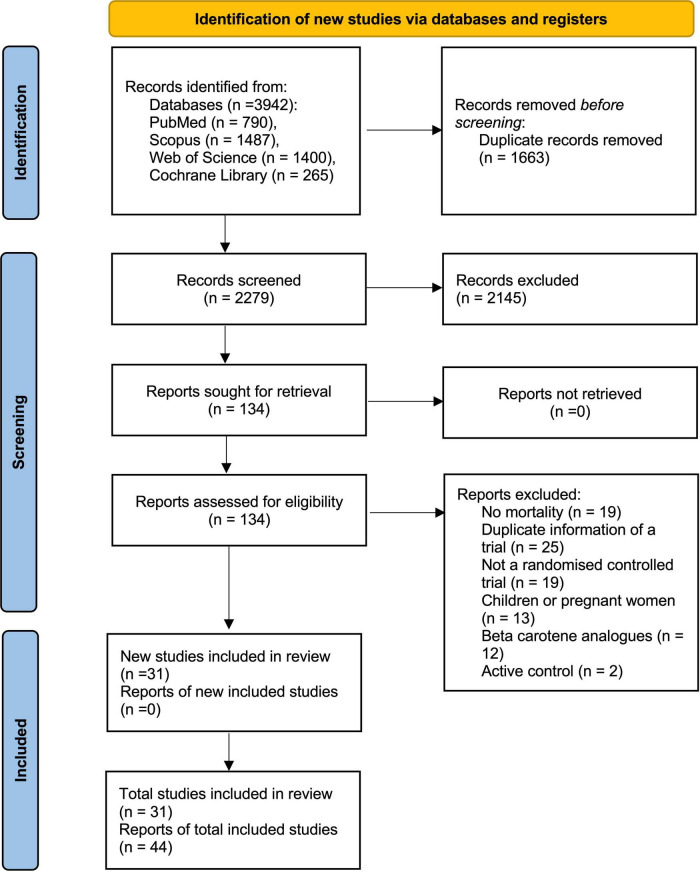
Search strategy and final included and excluded studies by the PRISMA flowchart.

We initially identified 3,942 records after searching databases and relevant bibliographies. After excluding 1,663 duplicated articles and 2,145 articles that did not satisfy the selection criteria, we reviewed the full texts of 134 articles and included 44 articles (27, 29, 30, 37–77) on 31 RCTs in the final analysis (Figure 1).

### Study Characteristics

Table 1 summarizes the characteristics of included trials, and Table 2 gives details of those trials. The final analysis comprised 216,734 participants, 108,622 in the beta-carotene supplement group and 108,112 in the placebo or no intervention groups, from 31 RCTs reported in 44 articles. In the studies in which age and gender were reported, the median age was 60.2 years (age range 32–85 years), and 49% of the subjects were women. The median treatment and follow-up periods were 3 and 4.6 years, respectively. There were 45,907 deaths, of which 4,609 deaths were from cancer, 3,796 deaths were from CVD, and 956 deaths were from cerebrovascular disease.

**TABLE 1:** Summary characteristics of included studies.

Characteristics	No. of trials (No. of participants)
**Eligible studies**	
Total No. of trials (No. of participants) Median (IQR) follow-up (years) Follow-up at least 4 years Median (IQR) No. of participants Total No. of deaths Median (IQR)% women Median (IQR) age (years)	31 (216,734) 4.6 (1.7–8.8) 16 (171,578) 382 (85, 5,883) 45,907 49 (15.45–58.44) 60.2 (54.2–67.7)
**Country**	
American European Asian-pacific	17 (119,297) 9 (63,937) 5 (33,500)

**TABLE 2:** Data summary of randomized controlled trials assessing the effects of beta-carotene supplementation on mortality (*n* = 44).

References	Country	Study characteristics	Treatment duration/follow-up period (median)	Intervention (dose)	Mortality cause	Intervention (death/total)	Control (death/total)
Albanes et al. (37)	Finland	*N* = 29,133 (mean age 57.2 y) Women: 0% Health status: smokers (5 + cigarettes/day) Design: 2.2 factorial	6.1/6.1 y	Beta-carotene (20 mg/d) + alpha-tocopherol (50 mg/d) vs. beta-carotene placebo	Colorectal cancer	23/14,560	23/14,573
Austin et al. (38)	Canada	*N* = 331 (median age 39.5 y) Women: 10.5% Condition: acquired immunodeficiency syndrome Design: Parallel	13/13 m	Beta-carotene (72 mg/d) + multivitamins and trace elements vs. beta-carotene placebo	HIV-related mortality	13/165	23/166
Bairati et al. (39)	Canada	*N* = 156 (mean age 62.5 y) Women: 21% Condition: stage I or II head and neck cancer Design: Parallel	3.1/6.5 y	Beta-carotene (30 mg/d) + alpha-tocopherol (400 UI/d) vs. placebo	All-cause	37/79	30/77
Blot et al. (40)	China	*N* = 29,450 (age range 40–69 y) Women: 55% Health status: at risk of esophageal/gastric cardia cancer Design: 2.2.2.2 factorial	5.25/5.25 y	Beta-carotene (15 mg/d) + vitamin E and selenium + micronutrients vs. beta-carotene placebo	Cancer Cerebrovascular disease	369/14,729 249/14,729	423/14,721 274/14,721
Brown et al. (41)	United States	*N* = 80 (mean age 53 y) Women: 13% Health status: coronary disease Design: 2.2 factorial	3/3 y	Antioxidant vitamins (beta-carotene 25 mg/d) vs. placebo	All-cause Cardiovascular cause	11/42 3/42	12/38 7/38
Chew et al. (42)	United States	*N* = 4,757 (median age 69 y) Women: 56% Health status: age-related eye disease Designs: 2.2 factorial	6.3/10 y	Beta-carotene (15 mg/d) + vitamin C (500 mg/d) + vitamin E (400 IU/d) ± zinc (80 mg/d) vs. placebo	All-cause	439/2,370	427/2,387
Chylack et al. (43)	United States	*N* = 297 (mean age 68 y) Women: 59% Condition: age-related cataract Design: Parallel	3/3 y	Antioxidant micronutrients (beta-carotene 18 mg/d) vs. placebo	All-cause	9/149	3/148
Garbagnati et al. (44)	Italy	N = 34 (mean age 66.75 y) Women: 44.5% Condition: stroke Designs: 2.2 factorial	1/1 y	Antioxidants (beta-carotene 19 mg/d) vs. placebo	Cardiovascular disease	1/16	3/18
Gaziano et al. (45)	United States	*N* = 14,641 (mean age 64.3 y) Women: 0% Health status: General population Design: 2.2.2.2 factorial	11.2/11.2 y	Beta-carotene (50 mg/alternate days) + multivitamins vs. beta-carotene placebo	All-cause Cancer	1,345/7,317 403/7,317	1,412/7,324 456/7,324
Girodon et al. (46)	France	*N* = 362 (mean age 83.9 y) Women: 74.58% Condition: Institutionalized elderly Design: 2.2 factorial	2/2 y	Vitamins (beta-carotene 6 mg/d) vs. placebo	All-cause	45/180	51/182
Goodman et al. (47)	United States	*N* = 18,314 (median age 58 y) Women: 34% Health status: Smoker or asbestos exposed Designs: Parallel	4/10 y	Beta-carotene (30 mg/d) + retinyl palmitate (25,000 IU/d) vs. placebo	All-cause Lung cancer Cardiovascular disease	1,855/9,420 294/9,420 354/9,420	1,509/8,894 227/8,894 319/8,894
Graat et al. (48)	Netherlands	*N* = 316 (mean age 73.2 y) Women: 48.5% Condition: non-institutionalized elderly Design: 2.2 factorial	15/15 m	Multivitamin-mineral capsule (beta-carotene 2.4 mg/d) vs. placebo	All-cause	0/163	5/153
Greenberg et al. (49)	United States	*N* = 1,805 (mean age 63.2 y) Women: 30% Health condition: Basal cell or squamous cell carcinoma Designs: Parallel	4.3/8.2 y	Beta-carotene (50 mg/d) vs. placebo	All-cause Cardiovascular disease Cancer	146/913 68/913 38/913	139/892 59/892 44/892
Grieger et al. (50)	Australia	*N* = 115 Women: 52% Health condition: aged care residents Designs: Parallel	6/6 m	Multivitamin (beta-carotene 3 mg/d) vs. placebo	All-cause	3/58	4/57
Heart Protection Study Collaborative Group (51)	United Kingdom	*N* = 20,536 (age range 40–80 y) Women: 24.74% Health status: coronary disease, occlusive arterial disease, or diabetes Design: 2.2 factorial	5/5 y	Antioxidant vitamins (20 mg/d beta-carotene) vs. placebo	All-cause Coronary heart disease Stroke	1,446/10,269 664/10,269 108/10,269	1,389/10,267 630/10,267 107/10,267
Alpha-Tocopherol, Beta Carotene Cancer Prevention Study Group (27)	Finland	*N* = 29,133 (mean age 57.2 y) Women: 0% Health status: Smokers (5 + cigarettes/day) Design: 2.2 factorial	6.1/6.1 y	Beta-carotene (20 mg/d) + alpha-tocopherol (50 mg/d) vs. beta-carotene placebo	Cancer Lung cancer	582/14,560 302/14,560	534/14,573 262/14,573
Heinonen et al. (52)	Finland	*N* = 29,133 (mean age 57.2 y) Women: 0% Health status: Smokers (5 + cigarettes/day) Design: 2.2 factorial	6.1/6.1 y	Beta-carotene (20 mg/d) + alpha-tocopherol (50 mg/d) vs. beta-carotene placebo	Prostate cancer	33/14,560	29/14,573
Hennekens et al. (29)	United States	*N* = 22,071 (mean age 53 y) Women: 0% Health status: General population Design: 2.2 factorial	12/12 y	Beta-carotene (50 mg/alternate days) + aspirin vs. beta-carotene placebo	All-cause Cardiovascular disease Malignant neoplasm	979/11,036 338/11,036 386/11,036	968/11,035 313/11,035 380/11,035
Hercberg (53)	France	*N* = 13,017 (mean age 49 y) Women: 60.5% Health status: General population Designs: Parallel	7.5/12.5 y	Antioxidant vitamins and minerals (beta-carotene 6 mg/d) vs. placebo	All-cause	156/6,481	178/6,536
Jiamton et al. (54)	Thailand	*N* = 481 (mean age 32 y) Women: 61% Health status: HIV-infected Designs: Parallel	48/48 w	Immunace Micronutrient supplement (beta-carotene 6 mg/d) vs. placebo	HIV-related mortality	8/242	15/239
Kataja-Tuomola et al. (55)	Finland	*N* = 1,700 (mean age 57.2 y) Women: 0% Health status: Smokers (5 + cigarettes/day) with diabetes Design: 2.2 factorial	6.1/6.1 y	Beta-carotene (20 mg/d) + alpha-tocopherol (50 mg/d) vs. beta-carotene placebo	Diabetes-related mortality	168/877	150/823
Lai et al. (56)	Finland	*N* = 29,133 (mean age 57.2 y) Women: 0% Health status: Smokers (5 + cigarettes/day) Design: 2.2 factorial	6.1/24 y	beta-carotene (20 mg/d) + alpha-tocopherol (50 mg/d) vs. beta-carotene placebo	Chronic liver disease	121/14,560	116/14,573
Lamas et al. (57)	United States	*N* = 1,708 (median age 65 y) Women: 18% Health status: Post myocardial infarction Design: 2.2 factorial	31/55 m	Multivitamin and multimineral mixture (beta-carotene 25,000 IU/d) + IV chelation infusions vs. placebo	All-cause Cardiovascular disease	87/853 45/853	93/855 56/855
Lee et al. (30)	United States	*N* = 39,876 (mean age 54.6 y) Women: 100% Health status: Healthy Design: 2.2.2 factorial	2.1/4.1 y	Beta-carotene (55 mg on alternate days) + aspirin and vitamin E vs. beta-carotene placebo	All-cause Cardiovascular disease Cancer	59/19,939 14/19,939 31/19,939	55/19,937 12/19,937 28/19,937
Leppälä et al. (58)	Finland	*N* = 28,519 (mean age 57.2 y) Women: 0% Health status: Stroke-free smokers (5 + cigarettes/day) Design: 2.2 factorial	6.1/6.1 y	Beta-carotene (20 mg/d) + alpha-tocopherol (50 mg/d) vs. beta-carotene placebo	Stroke	82/14,246	78/14,273
Li et al. (59)	China	*N* = 3,318 (mean age 54 y) Women: 56% Health status: Esophageal dysplasia Design: Parallel	6/6 y	Vitamins and minerals (15 mg/d beta-carotene) vs. placebo	All-cause Cancer Cerebrovascular disease	157/1,657 87/1,657 22/1,657	167/1,661 89/1,661 35/1,661
Lin et al. (60)	United States	*N* = 8,171 (mean age 60.4 y) Women: 100% Health status: High risk of cardiovascular disease Design: 2.2.2.2 factorial	9.4/9.4 y	Beta-carotene (50 mg every other day) + antioxidants vs. beta-carotene placebo	Cancer	80/4,084	96/4,087
Liu et al. (61)	Canada	*N* = 763 (mean age 85 y) Women: 70% Health status: Institutionalized elderly Design: Parallel	19/19 m	Multivitamin and multimineral (beta-carotene 16 mg/d) vs. placebo	All-cause	96/379	97/384
Margalit et al. (62)	United States	*N* = 383 (median age 73 y) Women: 0% Health status: Prostate cancer Design: 2.2 factorial	12/22.5 y	Beta-carotene (50 mg/alternate days) ± aspirin vs. placebo	Prostate cancer	20/192	25/191
Mayne et al. (63)	United Kingdom	*N* = 264 (mean age 68 y) Women: 19% Health status: Head and neck cancer Design: Parallel	4.25/4.25 y	Beta-carotene (50 mg/d) vs. placebo	All-cause	21/135	26/129
Papadimitrakopoulou et al. (64)	United States	*N* = 84 (mean age 56 y) Women: 48.9% Health status: Oral premalignancy Design: Parallel	3/5 y	Beta-carotene (50 mg/d) + retinyle palmitate vs. beta-carotene placebo	All-cause	1/47	0/37
Age-Related Eye Disease Study 2 Research Group (65)	United States	*N* = 4,203 (median age 74 y) Women: 56.75% Health status: AMD Design: 2.2 factorial	5/5 y	Macular xanthophylls (10 mg/d lutein + 2 mg/d zeaxanthin) + omega-3 fatty acids (350 mg/d DHA + 650 mg/d EPA) vs. macular xanthophylls placebo	All-cause	746/2,123 *^PC^*	727/2,080 *^PC^*
Pathak et al. (66)	India	*N* = 136 (median age 56 y) Women: 14.6% Health status: Advanced non-small cell lung cancer Design: Parallel	2/2 y	Antioxidants (60 mg/d beta-carotene) + chemotherapy vs. chemotherapy	All-cause	54/64	64/72
Plummer et al. (67)	Venezuela	*N* = 1,980 (mean age 35–69 y) Women: 52.7% Condition: Precancerous gastric lesions Design: Parallel	3/3 y	Antioxidant vitamins (beta-carotene 18 mg/d) vs. placebo	All-cause	16/990	11/990
Prince et al. (68)	United Kingdom	*N* = 61 (mean age 58 y) Women: 92% Health condition: primary biliary cirrhosis Design: Cross-over	12/12 w	Antioxidant supplementation (beta-carotene 3 mg/d) vs. placebo	Ischemic heart disease	1/29	0/32
Qu et al. (69)	China	*N* = 29,450 (age range 40–69 y) Women: 55% Health status: At risk of esophageal or stomach cancer Design: 2^4^ partial factorials	5.25/15.2 y	Beta-carotene (15 mg/d) + vitamin E and selenium vs. placebo	Liver cancer	68/14,729	83/14,721
Rautalahti et al. (70)	Finland	*N* = 29,133 (mean age 75.7 y) Women: 0% Health status: Smokers (5 + cigarettes/day) Design: 2.2 factorial	6.1/6.1 y	Beta-carotene (20 mg/d) + alpha-tocopherol (50 mg/d) vs. beta-carotene placebo	Pancreatic carcinoma	35/14,560	48/14,573
Richer et al. (71)	United States	*N* = 60 (mean age 75.3 y) Women: 5% Condition: Atrophic age-related macular degeneration Design: Parallel	12/12 m	Lutein (10 mg/d) vs. placebo	All-cause	1/29	2/31
Toma et al. (72)	Italy	*N* = 214 (median age 60.5 y) Women: 9.8% Health condition: Stage I-II head and neck cancer Design: Parallel	3/4.9 y	Beta-carotene (75 mg/d) vs. no treatment	All-cause Head and neck tumor	9/104 5/104	15/110 6/110
Törnwall et al. (73)	Finland	*N* = 29,133 (mean age 57.7 y) Women: 0% Health status: Smokers at risk of major coronary event Design: 2.2 factorial	6.1/6.1	Beta-carotene (20 mg/d) + alpha-tocopherol (50 mg/d) vs. beta-carotene placebo	Coronary heart disease	456/14,560	449/14,573
Virtamo et al. (74)	Finland	*N* = 29,133 (mean age 57.7 y) Women: 0% Health status: Smokers (5 + cigarettes/day) Design: 2.2 factorial	6.1/6.1 y	Beta-carotene (20 mg/d) + alpha-tocopherol (50 mg/d) vs. beta-carotene placebo	Urothelial cancer Renal cell cancer	13/14,560 16/14,560	11/14,573 25/14,573
Virtamo et al. (75)	Finland	*N* = 29,133 (mean age 57.7 y) Women: 0% Health status: Smokers (5 + cigarettes/day) Design: 2.2 factorial	6.1/14.1 y	Beta-carotene (20 mg/d) + alpha-tocopherol (50 mg/d) vs. beta-carotene placebo	All-cause	5,555/14,560	5,276/14,573
Wang et al. (76)	China	*N* = 29,450 (median age 52 y) Women: 55% Health status: At risk of esophageal/gastric cardia cancer Design: 2.2.2.2 factorial	5.25/30 y	Beta-carotene (15 mg/d) + vitamin E and selenium + micronutrients vs. beta-carotene placebo	All-cause	9,910/14,729	9,824/14,721
Wright et al. (77)	Finland	*N* = 29,133 (mean age 57.7 y) Women: 0% Health condition: Smokers (5 + cigarettes/day) Design: 2.2 factorial	6.1/6.1 y	Beta-carotene (20 mg/d) + alpha-tocopherol (50 mg/d) vs. beta-carotene placebo	Oral/pharyngeal cancer Esophageal cancer laryngeal cancer	10/14,560 6/14,560 5/14,560	7/14,573 9/14,573 5/14,573

*ATBC, Alpha-Tocopherol, Beta-Carotene Cancer Prevention Study; NIT1, Nutrition Intervention Trial (NIT); The General Population Trial; HATS, The HDL-Atherosclerosis Treatment Study; AREDS, Age Related Eye Disease Study; REACT, The Roche European American Cataract Trial; PHSII, Physicians Health Study; CARET, The Beta-Carotene and Retinol Efficacy Trial; SCPS, Skin Cancer Prevention Study; HPS, Heart Protection Study; PHS, Physicians Health Study; SUVIMAX, The Supplementation en Vitamines et Mineraux Antioxydants; WHS, Women’s Health Study; AREDS2, Age-Related Eye Disease Study 2; PC, personal contact; LAST, Lutein Antioxidant Supplementation Trial.*

The selected articles were published from 1993 through 2018, spanning 25 years. The countries in which the studies were conducted were as follows: United States (*n* = 13), Canada (*n* = 3), United Kingdom (*n* = 3), China (*n* = 2), France (*n* = 2), Italy (*n* = 2), Finland (*n* = 1), Netherlands (*n* = 1), Venezuela (*n* = 1), India (*n* = 1), Thailand (*n* = 1), and Australia (*n* = 1). The studies included healthy subjects (general population, physicians, and nurses); patients with oral premalignancy, skin, lung, and head and neck cancer; adults with underlying CVD or cerebrovascular diseases, and acquired immunodeficiency syndrome (AIDS), primary biliary cirrhosis, and age-related eye diseases; persons at risk of esophageal/gastric cardia cancer; smokers or asbestos-industry workers; and institutionalized elderlies.

Among the 31 trials, 30 had a placebo group, and 1 had a no-intervention group as the control (77). Further, 16 trials used the parallel design, 14 used the factorial design, and one study used a cross-over design (67). The following 3 trials were reported in 16 articles: the Alpha-Tocopherol Beta-Carotene Prevention Study (*n* = 11), Nutrition Intervention Trial; The General Population Trial (*n* = 3), and the Physicians’ Health Study (*n* = 2).

### Quality of the Included Trials

Supplementary Figures 1, 2 show the quality of the included trials. Twenty-four trials were classified as having a low risk of bias. The remaining 4 trials had one or more inadequate components (64, 66, 72, 76), and 1 trial had an unclear risk of bias (63). Supplementary Figures 3, 4 show the GRADE assessment of the quality. The overall results showed a high quality of the studies.

### Meta-Analysis of the Effect of Beta-Carotene Supplements on Mortality Risk

**FIGURE 2:**
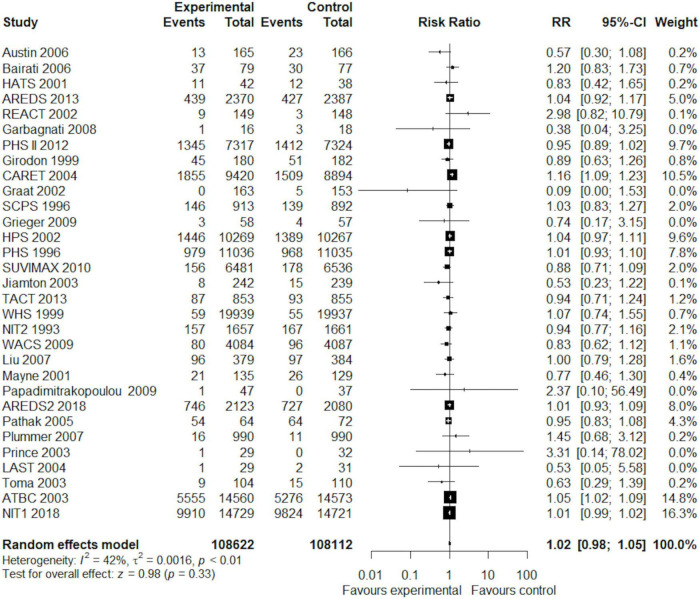
Forest plot showing the effect of beta-carotene supplementation on total mortality in 31 randomized controlled trials.

**FIGURE 3:**
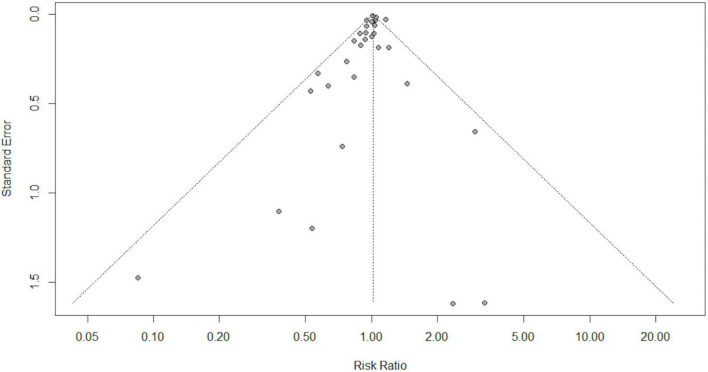
Funnel plot for publication bias in 31 randomized controlled trials.

Overall, in a random-effects model meta-analysis of all the 31 trials (27, 29, 30, 37–77), there was no statistically significant difference in total mortality between the beta-carotene supplementation group and the control group (RR 1.02, 95% CI 0.98–1.05, *I*^2^ = 42%; Figure 2). Funnel plot analysis showed no asymmetry (Figure 3); additionally, the Egger test (*P* = 0.25) and Begg’s test (*P* = 0.85) detected no significant small-study effects.

Subgroup analyses according to the number of participants, the number of events, gender, age groups, beta-carotene dose, follow-up duration, type of intervention (singly or combined beta-carotene supplements), participant health status, and the control group did not show any difference in total mortality among the participants (Table 3). Table 4 shows the results of the subgroup analyses on cause-specific mortality. Beta-carotene supplementation was not associated with cancer mortality (RR 0.98, 95% CI 0.90–1.07, *I*^2^ = 37%). However, the use of beta-carotene supplements significantly increased mortality among lung cancer patients (RR 1.14, 95% CI 1.02, 1.27, *I*^2^ = 3%). As for CVD mortality, we found no statistically significant difference between the groups (RR 1.04, 95% CI 0.98, 1.11, *I*^2^ = 0%). Similarly, beta-carotene supplementation did not reduce the risk of death from cerebrovascular disease (RR 0.94, 95% CI 0.82, 1.06, *I*^2^ = 0%). However, a significant beneficial effect of beta-carotene on mortality risk was observed in participants with human immunodeficiency virus (HIV) infection (RR 0.55, 95% CI 0.33, 0.92, *I*^2^ = 0%).

**TABLE 3:** Subgroup analyses of the effect of beta-carotene on total mortality.

Subgroup title	No. of trials	No. of participants	*I*^2^ (%)	Risk ratio (95% CI)	*P*-value
**Overall**	31	216,734	42.0	1.02 (0.98, 1.05)	0.3
**No of participants**					
≥1,000 <1,000	15 16	212,980 3,754	58.0 4.0	1.02 (0.98, 1.05) 0.93 (0.83, 1.04)	0.1 0.2
**No of events**					
≥100 <100	16 15	211,899 4,835	55.0 15.0	1.02 (0.99, 1.05) 0.88 (0.71, 1.09)	0.2 0.3
**Age (years)**					
≥65 <65	11 20	12,879 203,855	0.0 56.0	1.00 (0.94, 1.07) 1.02 (0.98, 1.06)	0.9 0.3
**Gender**					
Women Men Women and men	2 3 26	48,047 65,845 102,842	9.0 73.0 39.0	0.92 (0.72, 1.17) 1.01 (0.95, 1.08) 1.02 (0.97, 1.06)	0.5 0.8 0.5
**Daily dose equivalent (mg)**					
≥ 20 < 20	15 16	155,812 60,922	55.0 0.0	1.02 (0.97, 1.08) 1.01 (0.99, 1.02)	0.5 0.4
**Follow up**					
At least 4 years Less than 4 years	16 15	171,578 45,156	57.0 2.0	1.02 (0.98, 1.06) 0.94 (0.85, 1.04)	0.3 0.2
**Intervention**					
Beta carotene alone Combined	4 27	2,343 214,391	0.0 47.0	0.96 (0.79, 1.16) 1.02 (0.98, 1.05)	0.6 0.3
**Participant health status**					
Healthy Unhealthy	5 26	89,921 126,813	19.0 42.0	0.97 (0.91, 1.04) 1.03 (0.99, 1.07)	0.4 0.1
**Control group**					
Placebo No intervention	30 1	216,520 214	43 -	1.02 (0.99, 1.05) 0.63 (0.29, 1.39)	0.3 0.25

**TABLE 4:** Effects of beta-carotene supplements vs. placebo or no intervention on cause-specific mortality.

Mortality cause	No. of trials	Risk ratio (95% CI)	I2 (%)	Model used
Cancer	13	0.98 (0.90, 1.07)	37.0	Random effects
Colorectal cancer	2	0.97 (0.68, 1.38)	0.0	Random effects
Esophagus and stomach cancer	2	0.93 (0.82, 1.06)	0.0	Random effects
Prostate cancer	3	0.93 (0.73, 1.18)	0.0	Random effects
Lung cancer	5	1.14 (1.02, 1.27)*	3.0	Random effects
Lung cancer in smokers	2	1.14 (1.03, 1.27)*	0.0	Random effects
Lung cancer in mixed smokers and non-smokers	3	0.94 (0.74, 1.20)	0.0	Random effects
Urinary tract cancer	2	0.82 (0.55, 1.21)	0.0	Random effects
Pancreatic cancer	2	0.85 (0.62, 1.16)	0.0	Random effects
Other cancer	6	0.86 (0.70, 1.06)	0.0	Random effects
Cardiovascular disease	12	1.04 (0.98, 1.11)	0.0	Random effects
Cerebrovascular disease	5	0.94 (0.82, 1.06)	0.0	Random effects
HIV-related causes	2	0.55 (0.33, 0.92)*	0.0	Random effects
Non-cancer, non-vascular cause	5	1.04 (0.95, 1.14)	0.0	Random effects

*Statistically significant.

## Discussion

The current meta-analysis found that the administration of beta-carotene supplements had no preventive effect on total mortality, mortality from cancer, and vascular and non-vascular diseases. Furthermore, no association was found within subgroup meta-analyses based on the number of participants, the number of events, sex, age groups, beta-carotene dose, follow-up duration, type of intervention (singly or combined beta-carotene supplements), participant health status, and control group. However, beta-carotene supplementation was significantly related to an increased risk of lung cancer mortality (RR 1.14, 95% CI 1.02, 1.27, *I*^2^ = 3%, *n* = 5). The effects of beta-carotene supplementation on increased lung cancer incidence and mortality among smokers have already been described, and several possible biological mechanisms have been proposed. In general, beta-carotene supplementation has not been shown to positively impact cancer prevention. In a systematic review and meta-analysis, no effect of beta-carotene supplementation was observed on the incidence of the total, pancreatic, colorectal, prostate, breast, melanoma, and non-melanoma skin cancers. However, a significant harmful effect of beta-carotene supplementation on the incidence of lung and stomach cancers was observed in people supplemented with beta-carotene at 20–30 mg/day, in smokers and asbestos workers compared to placebo (78). Beta-carotene may act as a pro-oxidant in the presence of chronic oxidative stress such as smoking (79) and it may enhance the oxidative stress initiated by cigarette smoking and stimulate toxic effects in tissues (80). Our study also found significant inverse associations of beta-carotene supplementation with the risk of HIV-related mortality; however, this was reported in only two studies. This is in line with previous evidence illustrating that persons in all stages of HIV infection generally have low circulating levels of micronutrients, including carotenoids, and low micronutrient concentrations are correlated with HIV disease progression and mortality (38).

Overall, the findings of the present meta-analysis of RCTs are inconsistent with previous meta-analyses of observational studies suggesting beneficial effects from high dietary or circulatory beta-carotene-rich fruits and vegetables on all-cause and CVD mortality (25, 26). Intervention studies are commonly considered to provide conclusive answers, whereas observational studies represent a better picture of the real-world population. There are evident differences between the findings of published trials, which could be explained by population characteristics (general, ill, or at high-risk subjects), the different doses of supplementation (dietary levels or higher), which can be associated with harmful health effects (81), and the type of supplement (alone or in association). In this last condition, when subgroup analysis was performed, only 4 out of 31 studies reported the use of beta-carotene alone. Although no significant difference was found in all-cause mortality (*p* = 0.64), a very low heterogeneity was discovered among these studies (*I*^2^ = 3.60%) with a trend in reduced mortality with beta-carotene supplementation (RR = 0.95, 95% CI 0.74. 1.16, Supplementary Figure 5). Indeed, it appears that optimal effects may be obtained with a combination of nutrients at similar levels to a healthy diet. A single antioxidant, such as beta-carotene, given at high doses in subjects with a high risk of diseases, such as smokers and asbestos-exposed workers, might not have considerable benefits and can even have adverse outcomes (82). Another possible reason for the harmful effect in clinical trials involving beta-carotene may be attributed to the purified synthetic form (83, 84). The effective uptake of synthetic all-trans beta-carotene seems to make the synthetic form more suitable for efficient absorption. However, the fact that synthetic beta-carotene can change normal serum trans/cis ratios favoring the trans-isomer may lead to an unfavorable effect. The effects of using all-trans synthetic beta-carotene are still not well-understood (84). It is assumed that synthetic beta-carotene rather than natural mixed carotenoids may stimulate cancer formation (85). Ultimately, higher antioxidant intakes, including beta-carotene, are associated with a better diet quality, which indicates higher intakes of nutrients such as fibers, minerals, and flavonoids, and lower intakes of unhealthy nutrients.

The present study has several possible limitations. Firstly, in the majority of the studies, synthetic beta-carotene was used. Clinical consequences of using natural beta-carotene are not well-understood because RCTs have yet to be conducted. Additional trials are required to understand the differential results of synthetic beta-carotene as an alternative to natural beta-carotene. Secondly, the results were accompanied by some evidence of heterogeneity. However, the subgroup analyses were performed to overcome this problem, implying that some of the study and participant characteristics were possible sources of the heterogeneity in the data. Thirdly, the database sources did not include EMBASE. However, CENTRAL and Scopus include several articles from EMBASE as the original source.

Our study has several strengths, as well. We updated the association of beta-carotene with total mortality, assessed its effects on cause-specific mortality, and showed a significant inverse association between beta-carotene intake and HIV-related mortality. Second, because of no evidence of publication bias, the results have not been altered by this type of bias.

In conclusion, we found no evidence of an overall preventive effect of beta-carotene supplements on total, cancer, CVD, and cerebrovascular mortality risk in our meta-analysis of RCTs published over the past 25 years. Instead, beta-carotene supplementation increased the risk of lung cancer mortality but decreased the risk of HIV-related mortality. Surely more studies should be performed to better define this issue, by confirming or denying our results. Therefore, beta-carotene supplementation’s possible beneficial or harmful effects on mortality must not be overstated.

## Data Availability Statement

The raw data supporting the conclusions of this article will be made available by the authors, without undue reservation.

## Author Contributions

GC and SD conceived of the presented manuscript. SA, SM, MI, and GS analyzed each article and performed the data extraction independently. VC, MI, and SM drafted the method and result section with the input of GC and SD. GS and VC drafted the introduction and discussion section with the input of SA, GC, and SD. All authors discussed the results and contributed to the final manuscript.

## Conflict of Interest

The authors declare that the research was conducted in the absence of any commercial or financial relationships that could be construed as a potential conflict of interest.

## Publisher’s Note

All claims expressed in this article are solely those of the authors and do not necessarily represent those of their affiliated organizations, or those of the publisher, the editors and the reviewers. Any product that may be evaluated in this article, or claim that may be made by its manufacturer, is not guaranteed or endorsed by the publisher.

## Supplementary Material

Supplementary Figure 1 Quality of the included trials by using the Cochrane Collaboration risk of bias tool.

Supplementary Figure 2 Quality of the included trials by using the Cochrane Collaboration risk of bias tool in summary with the studies.

Supplementary Figure 3 The GRADE assessment of the study’s quality for the all-cause mortality outcome.

Supplementary Figure 4 The GRADE assessment of the study’s quality for the all-cause mortality outcome with beta carotene alone.

Supplementary Figure 5 Subgroup analysis by the type of supplement (alone or in association).

Supplementary Figure 6 PRISMA checklist.

Supplementary Figure 7 Subgroup analysis by country.

Supplementary Table 1 Publication bias evaluation of each study by using the Cochrane Collaboration risk of bias tool.
