# Supplementary figures and images for: Association Between Beta-Carotene Supplementation and Mortality: A Systematic Review and Meta-Analysis of Randomized Controlled Trials
Source: Front Med (Lausanne). 2022 Jul 19;9:872310. doi: 10.3389/fmed.2022.872310 (PMC9343755; doi:10.3389/fmed.2022.872310)

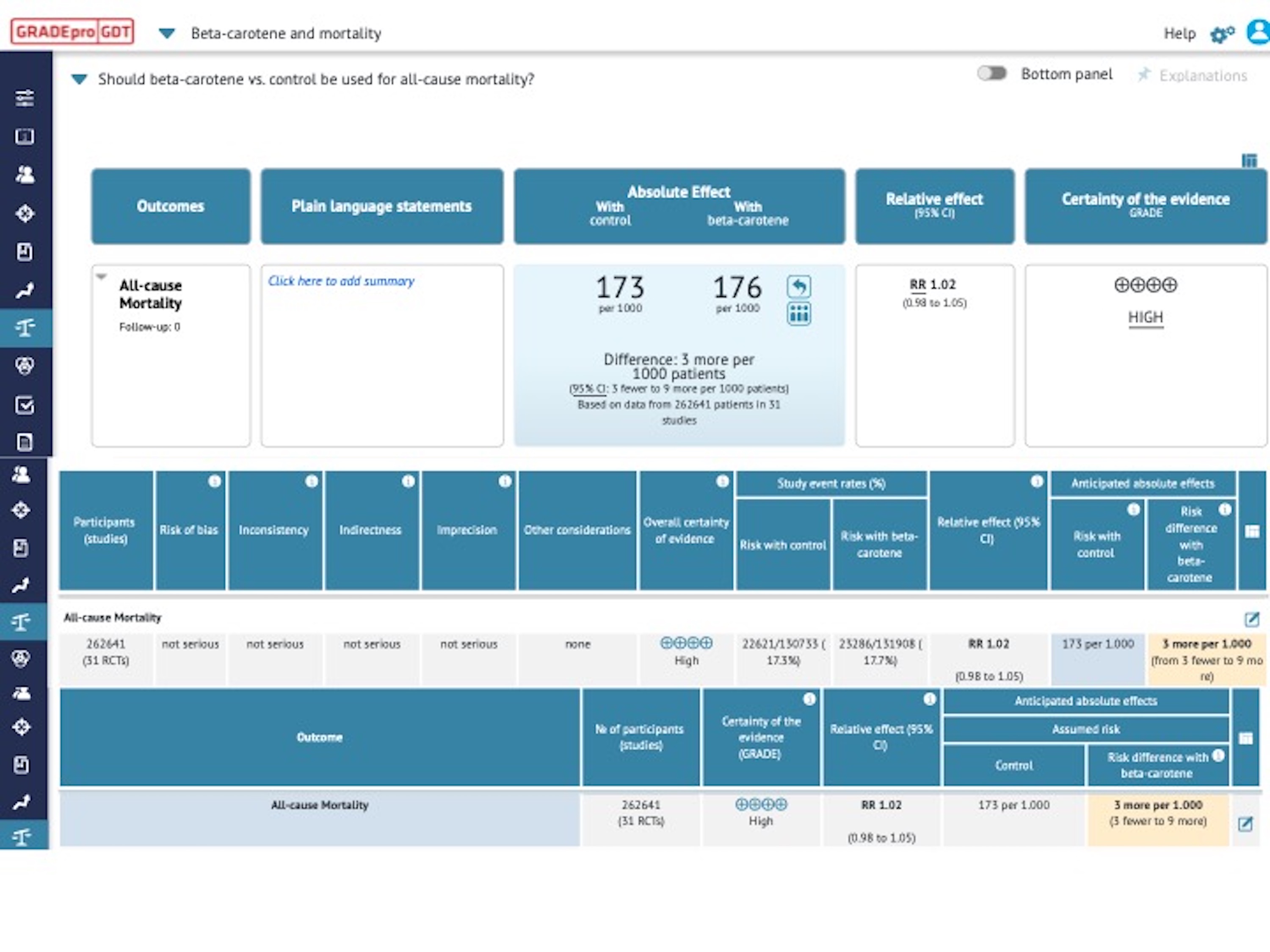

Supplement: Supplementary Figure 3 — The GRADE assessment of the study’s quality for the all-cause mortality outcome. [file Image_3.JPEG]

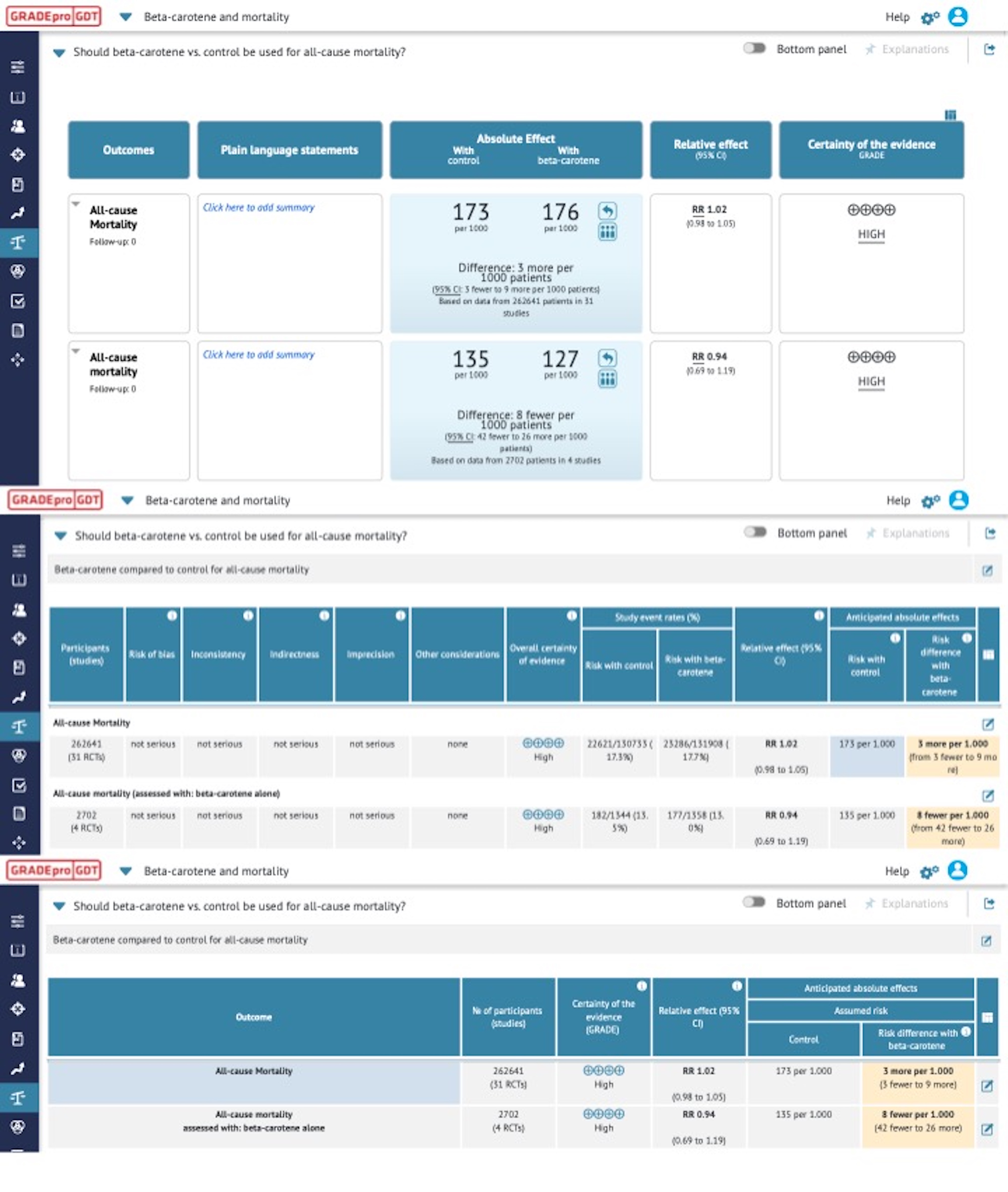

Supplement: Supplementary Figure 4 — The GRADE assessment of the study’s quality for the all-cause mortality outcome with beta carotene alone. [file Image_4.JPEG]

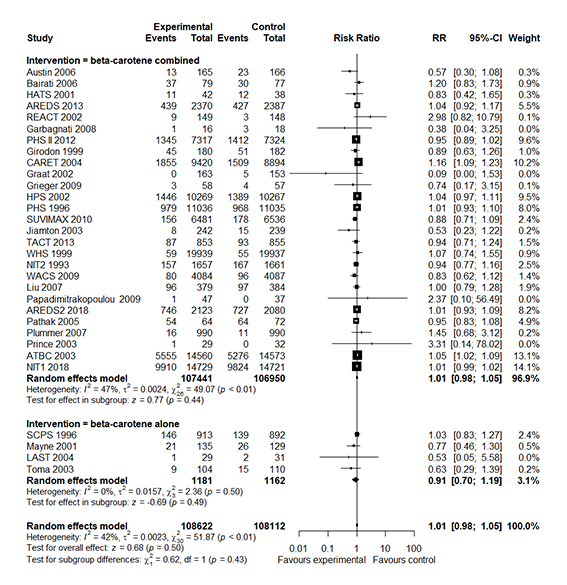

Supplement: Supplementary Figure 5 — Subgroup analysis by the type of supplement (alone or in association). [file Image_5.TIFF]

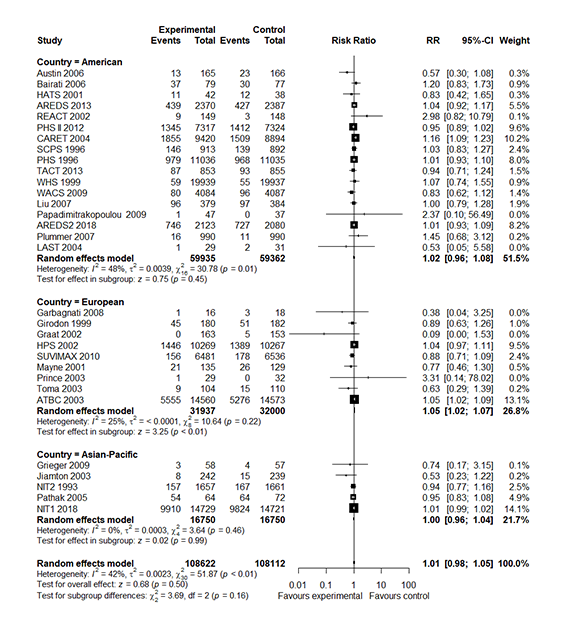

Supplement: Supplementary Figure 7 — Subgroup analysis by country. [file Image_7.tiff]
