# Supplementary material for: Association Between Beta-Carotene Supplementation and Mortality: A Systematic Review and Meta-Analysis of Randomized Controlled Trials
Source: Front Med (Lausanne). 2022 Jul 19;9:872310. doi: 10.3389/fmed.2022.872310 (PMC9343755; doi:10.3389/fmed.2022.872310)
Supplement: Supplementary Table 1 — Publication bias evaluation of each study by using the Cochrane Collaboration risk of bias tool. [file Table_1.pdf]

Studies	Random sequence generation	Allocation concealment	Blinding of participants and personnel	Blinding of outcome assessment	Incomplete outcome data (attrition bias)	Selective reporting
Austin 2006	Low	Low	Low	Low	Low	Low
Bairati 2006	Low	Low	Low	Low	Low	Low
HATS 2001	Low	Low	Low	Low	Low	Low
AREDS 2013	Low	Low	Low	Low	Low	Low
REACT 2002	Low	Low	Low	Low	Low	Low
De Klark 1998	Low	Low	Low	Low	Unclear	Low
Garbagnati 2009	Low	Low	Low	Low	Low	Low
PHS II 2012	Low	Low	Low	Low	Low	Low
Girodon 1999	Low	Low	Low	Low	Low	Low
CARET 2004	Low	Low	Low	Low	Low	Low
Graat 2002	Low	Low	Low	Low	Low	Low
SCPS 1996	Low	Low	Low	Low	Low	Low
Grieger 2009	Low	Low	Low	Low	Low	Low
HPS 2002	Low	Low	Low	Low	Low	Low
PHS 1996	Low	Low	Low	Low	Low	Low
SUVIMAX 2010	Low	Low	Low	Low	Low	Low
Jiamton 2003	Low	Low	Low	Low	Low	Low
TACT 2013	Low	Low	Low	Low	Low	Low
WHS 1999	Low	Low	Low	Low	Low	Low
NIT2 1993	Low	Low	Low	Low	Low	Low
WACS 2009	Low	Low	Low	Low	Low	Low
Liu 2007	Low	Low	Low	Low	Low	Low
Mayne 2001	Unclear	Unclear	Low	Low	Low	Low
Papadimitrakopoulou 2009	Unclear	Unclear	High	Low	Low	Low
AREDS2 2018	Low	Low	Low	Low	Low	Low
Pathak 2005	Low	Low	High	Low	Low	Low
Phillips 2006	Low	Low	High	Low	Low	Low
Plummer 2007	Low	Low	Low	Low	Low	Low
Prince 2003	Low	Low	Low	Low	Low	Low
LAST 2004	Low	Low	Low	Low	Low	Low
Toma 2003	Low	Low	High	Low	Low	Low
ATBC 2003	Low	Low	Low	Low	Low	Low

NIT1 2018	Low	Low	Low	Low	High	Low

Suppl Table 1
